# Oral Health of Lipjan Convicts: Kosovo Prison House

**DOI:** 10.1155/2018/6529658

**Published:** 2018-02-13

**Authors:** Luljeta Zajmi, Agim Begzati, Milaim Sejdini, Nora Berisha, Lumnije Krasniqi

**Affiliations:** ^1^Lipjan Correctional Center, Family Medicine Center Prishtina, Prishtina, Kosovo; ^2^Department of Pediatric and Preventive Dentistry, University Dentistry Clinical Center of Kosovo, Prishtina, Kosovo; ^3^Department of Orthodontics, University Dentistry Clinical Center of Kosovo, Prishtina, Kosovo

## Abstract

**Objectives:**

The oral health services of the prison population are considered more complex than those of the general population. The aim of this study was to examine the oral health status (the DMFT index and OHI index) and to evaluate the relation between the oral health and risk factors of inmates of this population, thus identifying the dental health status of inmates by gender, age, and the duration of their sentence.

**Materials and Methods:**

Our study has included a total number of 150 inmates, of both genders, from Lipjan prison house in Kosovo.

**Results:**

Oral health condition of inmates in Lipjan prison house is severe; the average value of DMFT is 8.44: for minors 6.22, while for adults 9.55. The assessment of DMFT index within the recruited inmates in our study shows that the mean rate of oral cure was 3.21, while the mean extraction value and caries were 3.55 and 3.58, respectively. The mean plaque test value was 1.44.

**Conclusion:**

Based on this research, we have concluded that the oral health condition of the inmates in Lipjan prison is not good, due to the presence of different risk factors among them.

## 1. Introduction

Providing dental services within the prison environment can make dentists face up with many unique challenges, including concerns about the threats to personal security, inability to move freely, and the requirements to deliver dental services that, in many cases, require modernization [1]. The oral health services of the prison population are more comprehensive than those of the general population [2]. The strengthening of oral health services among the prison population is a priority determined specifically by the WHO taking into account the increased prevalence of oral disease and limitations of providing the oral health services in the prison conditions [3].

The most important principle for better dental services in prison houses is the oral health promotion and more effective commitments towards dental treatment [4].

The health system in prison houses is organized in order to respond to the health needs of convicts. Health care in prison houses includes the following: primary health care, clinical problems, infectious diseases, mental health in prisons, oral health, and special health requirements for female inmates.

So far, no scientific research has been conducted in terms of oral health in Kosovo prison houses, but in the routine annual reports performed in our country, the problem of limited oral health services within the general health evaluations in prison houses has been brought up. According to several published studies in other countries, we can see that the oral health of convicts is accompanied by several barriers compared to the general population [5, 6]. Osborn et al. stated that “oral health care in prisons is low; therefore, measures shall be taken to promote and improve oral health in prisons” [7]. “Inmates have poorer general and oral health than the nonprison population,” concluded Heidari et al. [8]. Many people who suffer conviction are usually unemployed prior to their imprisonment, with poor financial incomes and lower social status. As it has been stated by this author, sixty-two percent (*n* = 76) of the study subjects were unemployed prior to their arrest, whereas the rest of them said that the most common previous employments were as a builder, a painter, a decorator, or in catering services [8].

In such circumstances, it is evident that their health in general is considerably neglected, especially oral health. Furthermore, the majority of convicts who enter prison suffer from poor oral health. Alcohol, tobacco smoking, and the use of drugs contribute to dental caries and oral mucosa disease [9]. According to the recently published data, the oral health condition of inmates is in a very severe condition [7, 8]. The need for dental services continues to grow with the increasing number of inmates. Current available literature indicates that the index of oral status amongst convicts is much worse than in the general population. Risk factors in prison houses increase due to the prevalence of drugs, tobacco, and low educational level. The drug addicts (e.g., heroin) mainly have xerostomia which precedes the rapid increase of caries lesions [10]. The inmates have had a few previous dental interventions; several convicts have physical and mental problems, and the majority experience anxiety when being examined by the dentist.

In a research conducted in 2003 in Australia, out of 789 inmates (657 males and 132 females), the average DMFT value was 20.4 [7]. In a research conducted in the USA, in a Correctional Service of Michigan, out of 251 males, within the inmates of 18–34 age groups, the DMFT value was 11.52 [11, 12].

In the outpatient department of Lipjan-Kosovo prison house, the dental clinic is supplied with all the necessary dental equipment, instruments, and materials, and it hires one general dentist.

Furthermore, this dental clinic has also been providing dental services to the convicts and detained subjects from other prison houses and detention centers such as Pristina and Prizren centers (Kosovo).

The aim of this descriptive epidemiologic study is to introduce the oral health status of the convicts in Lipjan prison (Kosovo).

The aim of this study was to examine the dental health status (the DMFT index) and to evaluate the relation between the oral health and the risk factors of inmates within this population. Moreover, the survey tends to identify the dental health status of the inmates by gender, age, and the duration of their sentence.

## 2. Materials and Methods

Our study involved a total number of 150 inmates, of both genders, from Lipjan prison house. Out of this number, 50 were juvenile male inmates, 50 were adult male inmates, and 50 were female inmates. The inmates were consecutively enrolled in order to reach the sample size of 150 individuals. For the comparison purposes of the results, we have divided the inmates/detainees by these respective age groups: 19–24 years old, 25–34 years old, and >35 years old. The patients were all informed about the purpose and the tasks of this research. The patients who refused to be examined have been replaced by other patients. The research did not include inmates and patients with chronic generalized parodontopathy, mobile prosthetic appliance users, remaining roots in both dental arches, and semi-impacted teeth.

The research included the following:

1. The evaluation of dental health status with the DMFT index, amongst the convicts in the Lipjan prison house
2. Separate DMFT structure specification for the number of carious/decayed teeth (D), extracted/missed (M) teeth, and filled (F) teeth
3. Assessment of the oral hygiene using the plaque index and the tartar index
4. Comparison of the values within the gender and the age of the inmates
5. Assessment of the inmates' oral health depending on the duration of their sentence
6. Proposal for the measures to improve the overall oral health

The assessment of the inmates' oral health has been conducted in the dental treatment center of the outpatient clinic of the Lipjan prison house, using dental mirrors, probes, and artificial light. An approval from the prison's directorate was obtained prior to the commencement of the research, and ethical principles of the Helsinki convention were implemented. The examination was carried out by the dentist working in the dental treatment center and professionally, with intra-examiner reliability of kappa = 0.95 based on the examination of 15 inmates of different ages, supported by the professors from the Faculty of Dentistry of the University of Prishtina, Kosovo. The manuscript has been approved by the ethical authority of Kosovo correctional service (08 No. 1249).

Data collection was completed utilizing questionnaires prepared for the purposes of this study that include the following data:

- General data (name and surname, birthplace, gender, age, nationality, education, smoking, drugs, alcohol, bruxism, breathing through the mouth, the sentence duration within the prison, teeth cleaning techniques, and brushing)
- Data regarding the dental status—the presence of caries lesions, fillings, and extraction due to caries (DMFT index)
- Assessment of the oral hygiene index

### 2.1. DMFT Index

DMFT index determination is conducted according to the presence of caries/decay (D), filling (F), and extraction (missing) due to the caries tooth (M). Decayed lesion cavity is defined as the presence of caries; it is visible for the eye, and there is a loss of transparency or dental probe stumble during easy probe.

### 2.2. Index of Oral Hygiene (OHI Index)

Evaluation of oral hygiene index is determined by using the Green Vermilion index for dental plaque and tooth tartar.

### 2.3. Data Analysis

The introduction of the data is completed by using the tables and graphs. Data processing is conducted with the statistical package Inst 3. The minimum and the maximum values were calculated from the statistical parameter index structure, arithmetic mean, and standard deviation. For the data testing, the Fisher exact test, χ^2^ test, Mann–Whitney *U* test, and Kruskal–Wallis test were used, and the significant difference of *P* < 0.05 was used.

## 3. Results

### 3.1. DMFT Index Assessment Results

The research included 150 inmates who were divided into two groups of 50 juvenile inmates or 100 adult inmates.  Table 1 shows the mean DMFT results for the whole number of examined inmates (8.65 (SD ± 5.54)). For juvenile inmates, the DMFT was 6.22 (SD ± 4.65), while it was 9.55 (SD ± 6.21) for adult inmates.

The average value of DMFT for adult inmates, according to their group age and gender, is presented in Table 2. The average DMFT of age group 19–24 years old was 7.48 (SD ± 4.93), that of 25–34 years old was 9.35 (SD ± 6.65), and that of 35+ years old was 12.75 (SD ± 5.86). The average value of DMFT for female inmates was 8.72 (SD ± 6 : 56). The DMFT average value of male inmates was 10.38 (SD ± 5.78).

Table 3 shows the average decayed teeth in the DMFT structure. The average value of decayed teeth (D) of the adult inm*a*tes between 19 and 24 years was 3.48 (SD ± 3.62); for those aged 25–34 years, the value was 3.23 (SD ± 3.92); and for those aged 35+ years, it was 2.33 (SD ± 2.81). The average value of D of the female inmates was 1.88 (SD ± 3.01). The average value of D of the male inmates was 4.32 (SD ± 3.71).

The average value for extracted teeth (missing due to caries, M) is introduced in Table 4. This value for adult inmates between the ages of 19 and 24 years was 21.2 (SD ± 2.18), for those aged 25–34 years was 3.07 (SD ± 3.29), and for those aged 35+ years was 6.33 (SD ± 4.11). The average value of M of female inmates was 3.64 (SD ± 3.96). The average value of M of male inmates was 3.50 (SD ± 3.13).

Table 5 shows the average of treated teeth (filled, F). The average value of F for the adult inmates between the ages of 19 and 24 years was 1.91 (SD ± 2.27), for those of the age group 25–34 years was 3.07 (SD ± 3.50), and for those aged 35+ years was 4.08 (SD ± 3.44). The average value of F for female inmates was 3.26 (SD ± 3.42). The average value of F for the male inmates was 2.60 (SD ± 2.98).

DMFT index value results based on the sentence duration of the adult inmates are presented in Table 6. For adult inmates who had a sentence till 1 year, the DMFT was 9.54 (SD ± 6.72); for those sentenced from 1 to 5 years, the DMFT was 8.58 (SD ± 4.38); and for those sentenced for more than 5 years, it was 6.83 (SD ± 4.45). As shown in Table 6, in between values of DMFT, D, M, and F according to the sentence duration using Kruskal–Wallis test, we found a significant statistical difference for *P* < 0.05.

### 3.2. Oral Hygiene Index Results (Plaque Index)

The average value of the dental plaque index/oral hygiene index (Table 7) of adult inmates for the age groups of 19–24 years was 1.58 (SD ± 0.56), for those of the age group 25–34 years was 1.28 (SD ± 0.63), and for those aged 35+ years was 1.54 (SD ± 0.88). The average value of the dental plaque index for female inmates was 1.36 (SD ± 0.69). The average value of the dental plaque index for male inmates was 1.52 (SD ± 0.68).

OHI value results according to the sentence duration are presented in Table 8. The total plaque index value for adult inmates was 1.44 (SD ± 0.69), the tartar index value was 0.41 (SD ± 0.59), and the OHI value was 1.85 (SD ± 1.10). Based on calculated values introduced in Table 8 between the values of plaque index, the tartar tooth index, and OHI according to the sentence duration using Kruskal–Wallis test, we found an important statistical significance for *P* < 0.05.

### 3.3. The Harmful Habits of the Inmates

Out of the overall number of juvenile inmates, 60% were tobacco users and 10% of the juvenile inmates and 8% of the adult inmates stated that they drink alcohol.

Furthermore, 8% of the juvenile inmates stated that they use drugs. While among adult inmates, 16% stated “yes” for taking drugs. Bruxism is very common among inmates; about 50% have confirmed that grinding teeth is their habit. Oral breathing is more common among inmates. Thus, it is expressed in 28% of juvenile inmates and 19% of adult inmates.

## 4. Discussion

Inmates in jails and prisons are considered as a high-risk population and are exposed to a high prevalence of different communicable diseases, mental disease, and chronic diseases, and they are exposed to a higher risk for different infective diseases. Due to these circumstances at the prisons, this vulnerable group of the population does not have any favorable conditions for the maintenance of their oral health, and realistically, their oral health needs are higher compared to the general population [13].

Oral health conditions of Lipjan prison house inmates are severe, and the average value of DMFT is 8.44, for minors 6.22, while for adults 9.55. Higher value of DMFT is recorded for older inmates (+35 years old: 12.75).

Comparing the oral health status of the inmates in Lipjan prison house with the oral health conditions in prisons around the world, there were no significant statistical differences.

A research similar to ours with an approximate mean of DMFT for adult inmates conducted in America, Correctional Service of Michigan, by Ormes, Carolyn, Thompson, and Brimm (1997) with a total number of 251 male inmates, for the age group 18–34 years, had the mean value of DMFT 11.52. We must note that most of these inmates were African-Americans [11, 14]. This mean DMFT is approximate with the DMFT of Lipjan male inmates being 10.55. High DMFT values (12.75) were found in the age group +35 years. Low DMFT (7.48) was found in the age group 19–24 years. But, we have found different values from the literature, sometimes very high; for example, in Australia, in a research conducted in 2003 on 789 inmates (657 males and 132 females) from 27 correctional centers in New South Wales, Australia, the mean DMFT value was 20.4 [7]. The mean DMFT index value of 340 inmates in South Africa reported by Naidoo et al. [15] was 15.45, while for 127 male Brazilian inmates the DMFT index value was 19.72 [16].

In Lipjan (Kosovo) prison, of 150 examined inmates, 50 were females, with the mean DMFT of 8.72. Compared to the data from the literature, these values are similar. In the research conducted for the purpose of determining the oral health of female inmates in the Correctional Department in New York City-Correction Department in Riker's Island Correctional Facility, out of the 183 inmates examined the DMFT mean value was 9.9 [17].

In our research, in order to determine the oral health of women in Lipjan prison house, we calculated a DMFT mean value of 8.5, and there was no significant difference compared to the female inmates in the Correctional Department of NY.

The evaluation of DMFT of inmates recruited in our study shows that the mean rate of oral cure was 3.21, while the mean extraction value and caries were 3.55 and 3.58, respectively. The main difference between our study and other results is that dental oral cure in our survey is 30%, while Ormes et al. [14] show a treatment success rate of over 60%.

During the comparison between the values of DMFT, D, M, and F and the sentence duration with the Kruskal–Wallis test, we have found that DMFT index values decrease with the increasing residence time in prison. Even though the value remains high, an improvement of oral health status has been noted. The data analysis according to the sentence duration shows that the number of treated teeth is the highest. Also, the plaque index is decreasing. This, as an overall result, is a consequence of active work of the prison dental clinic.

However, in our research through the OHI, the oral health status results as being in a quite severe condition. The total plaque index for adult inmates was 1.44. The author found some approximation between the data of the OHI of the inmates (1.96) and our data as well [18].

The data from the literature also reveal the negligence on oral hygiene: 97.4% of the subjects (inmates) needed oral hygiene instructions [19].

Bad habits such as smoking tobacco and the use of alcohol and drugs within the inmates were very commonly expressed. These bad habits can affect the overall situation of the oral health status of the inmates. The data from the literature also reveal a very high consumption of tobacco within the prison houses. In a study conducted by Akaji and Folaranmi, 52.2% of inmates were current smokers [20].

## 5. Conclusion and Recommendations

Based on this research, we have concluded that oral health conditions of the inmates in Lipjan prison house are not so good, due to the low structure of dental cure (approximately 30%) compared to the rate of dental extraction and dental caries (around 70% of DMFT structure).

Detention centers in Prishtina, Lipjan, and Prizren have no dental services; therefore, the inmates have to be transported to the dentist who works in Lipjan prison. This procedure in itself contains many difficulties. Detainees may be from different categories, including those of the high-risk category.

On the basis of this research, it is proved that the lack of dental services within the detention centers aggravates even more the already serious situation of the inmates' oral health. Taking into account the difficulties that a lot of doctors encounter in prisons, by security restrictions and by a large number of inmates, the need for further promotion of oral health in prisons is obvious.

The values of DMFT and OHI for inmates according to their sentence duration had some improvements. This means that the inmates who have remained in prison for a longer period of time, thanks to the available dental services, had experienced improvements of their oral health status.

We learn from the current available literature that the oral health condition of inmates is more serious than that within the general population. On the basis of this research, we found that there is a serious health condition within the inmates, but the serious oral health condition was evident in the general public as well.

The main aggravating factor of oral health for inmates is the low index of oral hygiene and then the bad habits of this population with direct impact on DMFT values, the plaque test, and the periodontal index. The presence of the dental clinic within the prison premises had resulted in an increased interest of inmates for dental treatment, for better oral hygiene, and alike. However, the DMFT and the plaque index values remain high, so inmates' health care should be more active.

### 5.1. Recommendations

There is a need for an increased awareness within our society of the wider population regarding oral health issues. Therefore, it is an imperative for the health care decision-makers to provide more dental services within the detention centers of our country and to further promote the oral health in prisons. The existence of full-service dental clinics within prisons will significantly affect the improvement of the convicts' health status.

Overall, the promotion of oral health in prisons, better health education, and the improvement of bad habits should be of top priority.

The measures at the central government level regarding the serious condition of the oral health of our inmates and of the general population need to be very serious and always in compliance with the WHO criteria.

## Figures and Tables

**Table 1:** DMFT values according to the groups.

DMFT	Group		Total
	Juvenile inmates	Adult inmates	
*N*	50	100	150
Mean	**6.22**	**9.55**	**8.65**
SD	4.65	6.21	5.54
Min	0	0	0
Max	26	28	28
Mann–Whitney *U* test	*U*′ = 1304, *P*=0.712	*U*′ = 5552, *P*=0.177	

**Table 2:** DMFT values for adult inmates according to their age group and gender.

Gender	DMFT	Age group/mean age			Total
		19–24 (21.5 ± 1.7)	25–34 (28.7 ± 2.6)	35+ (40.2 ± 6.2)	
	*N*	14	22	14	50
F	Mean	6.21	7.18	13.64	**8.72**
	SD	6.13	5.34	6.46	6.56
	*N*	19	21	10	50
M	Mean	8.42	11.62	11.50	**10.38**
	SD	3.73	7.24	4.95	5.78
	*N*	**33**	**43**	**24**	**100**
Total	Mean	**7.48**	**9.35**	**12.75**	**9.55**
	SD	**4.93**	**6.65**	**5.86**	**6.21**

**Table 3:** Decay (D) value of the adult inmates according to the age group and gender.

Gender	D	Age group/mean age			Total
		19–24 (21.5 ± 1.7)	25–34 (28.7 ± 2.6)	35+ (40.2 ± 6.2)	
	*N*	14	22	14	50
F	Mean	2.86	1.55	1.43	1.88
	SD	4.52	1.74	2.74	3.01
	*N*	19	21	10	50
M	Mean	3.95	5.00	3.60	4.32
	SD	2.84	4.76	2.50	3.71
	*N*	**33**	**43**	**24**	**100**
Total	Mean	**3.48**	**3.23**	**2.33**	**3.10**
	SD	**3.62**	**3.92**	**2.81**	**3.58**

**Table 4:** Missing (M) values of adult inmates according to age group and gender.

Gender	M	Age group/mean age			Total
		19–24 (21.5 ± 1.7)	25–34 (28.7 ± 2.6)	35+ (40.2 ± 6.2)	
	*N*	14	22	14	50
F	Mean	1.50	2.64	7.36	**3.64**
	SD	1.61	3.26	4.24	3.96
	*N*	19	21	10	50
M	Mean	2.74	3.52	4.90	**3.50**
	SD	2.42	3.34	3.67	3.13
	*N*	**33**	**43**	**24**	**100**
Total	Mean	**2.21**	**3.07**	**6.33**	**3.57**
	SD	**2.18**	**3.29**	**4.11**	**3.55**

**Table 5:** Filled (F) value of the adult inmates according to age group and gender.

Gender	F	Age group/mean age			Total
		19–24 (21.5 ± 1.7)	25–34 (28.7 ± 2.6)	35+ (40.2 ± 6.2)	
	*N*	14	22	14	50
F	Mean	2.00	3.05	4.86	**3.26**
	SD	1.75	3.88	3.48	3.42
	*N*	19	21	10	50
M	Mean	1.84	3.10	3.00	**2.60**
	SD	2.63	3.14	3.23	2.98
	*N*	**33**	**43**	**24**	**100**
Total	Mean	**1.91**	**3.07**	**4.08**	**2.93**
	SD	**2.27**	**3.50**	**3.44**	**3.21**

**Table 6:** DMFT, D, M, and F values according to the sentence duration.

Sentence duration	*N*	DMFT		D		M		F	
		Mean	SD	Mean	SD	Mean	SD	Mean	SD
Till 1 year	68	9.54	6.72	3.97	3.88	3.01	3.85	2.62	3.08
1–5 years	26	8.58	4.38	2.46	1.88	2.65	2.53	3.50	3.35
Over 5 years	6	6.83	4.45	0.33	0.52	2.50	3.33	4.00	4.00
Total	100	9.55	6.21	3.10	3.58	3.57	3.55	2.93	3.21
Kruskal–Wallis test		*P* < 0.05		*P* < 0.05		*P* < 0.05		*P* < 0.05	

**Table 7:** Dental plaque index value of adult inmates according to their age group and gender.

Gender	Plaque index	Age group/mean age			Total
		19–24 (21.5 ± 1.7)	25–34 (28.7 ± 2.6)	35+ (40.2 ± 6.2)	
	*N*	14	22	14	50
F	Mean	1.43	1.23	1.50	**1.36**
	SD	0.65	0.61	0.85	0.69
	*N*	19	21	10	50
M	Mean	1.68	1.33	1.60	**1.52**
	SD	0.48	0.66	0.97	0.68
	*N*	**33**	**43**	**24**	**100**
Total	Mean	**1.58**	**1.28**	**1.54**	**1.44**
	SD	**0.56**	**0.63**	**0.88**	**0.69**

**Table 8:** Plaque and tartar index values according to the sentence duration.

Sentence duration	*N*	Plaque index		Tartar index		Total OHI	
		Mean	SD	Mean	SD	Mean	SD
Till 1 year	68	1.56	0.76	0.51	0.63	2.07	1.20
1–5 years	26	1.23	0.43	0.23	0.43	1.46	0.65
Over 5 years	6	1.00	0.00	0.00	0.00	1.00	0.00
Total	**100**	**1.44**	**0.69**	**0.41**	**0.59**	**1.85**	**1.10**
Kruskal–Wallis test		*P* < 0.05		*P* < 0.05		*P* < 0.05	
